# Measurement of IgE to purified sesame storage proteins in sera from sesame allergic Japanese children

**DOI:** 10.1186/2045-7022-1-S1-P2

**Published:** 2011-08-12

**Authors:** Peter Brostedt, Sigrid Sjölander, Cecilia Ericson, Anette Holtz, Raimo Carlsson, Komei Ito

**Affiliations:** 1 Phadia AB, Uppsala, Sweden; 2 Aichi Children's Health and Medical Center, Department of Allergy, Obu, Japan

## Background

The storage proteins from sesame seed, 11S globulin, 7S vicilin and 2S albumin, are official WHO/IUIS allergens. However, little is known about the IgE reactivity to the individual sesame allergen components.

## Objective

To investigate the IgE reactivity to purified sesame storage proteins in sera from sesame allergic Japanese children.

## Methods

The three storage proteins were all purified from unpolished sesame seeds extracted at pH 7,5. The 11S globulin and the 7S vicilin were enriched by precipitation in water. Pure 11S globulin was obtained by a further precipitation with 35% ammonium sulfate followed by anion exchange chromatography. Pure 7S vicilin was obtained by adding gel filtration and anion exchange chromatography. The 2S albumin was purified by cation exchange chromatography and gel filtration. Identities were confirmed by MALDI-TOF analysis. The three purified storage proteins were conjugated to the ImmunoCAP^®^ matrix and quantitative IgE responses in sera from five sesame allergic Japanese children were measured.

## Results

All the sera from sesame allergic Japanese children showed specific IgE reactivity to sesame extract. Out of the five sera, four had IgE response to 11S globulin, all five to 7S vicilin and three to 2S albumin.

## Conclusions

IgE reactivity to all three storage proteins in sesame seed, 11S globulin, 7S vicilin and 2S albumin, was shown in sera from the sesame allergic Japanese children.

